# Differentiating False Loss of Resistance from True Loss of Resistance While Performing the Epidural Block with the CompuFlo® Epidural Instrument

**DOI:** 10.1155/2019/5185901

**Published:** 2019-02-03

**Authors:** Pasquale Vaira, Michela Camorcia, Tiziana Palladino, Matteo Velardo, Giorgio Capogna

**Affiliations:** ^1^Department of Anesthesiology, Casa Sollievo della Sofferenza Hospital, San Giovanni Rotondo, (FG), Italy; ^2^Department of Anesthesiology, CdC Città di Roma, Rome, Italy; ^3^European School of Obstetric Anesthesia, EESOA, Rome, Italy

## Abstract

**Background:**

The occurrence of false losses of resistance may be one of the reasons for inadequate or failed epidural block. A CompuFlo® epidural instrument has been introduced to measure the pressure of human tissues in real time at the orifice of a needle and has been used as a tool to identify the epidural space. The aim of this study was to investigate the sensitivity and the specificity of the ability of CompuFlo® to differentiate the false loss of resistance from the true loss of resistance encountered during the epidural space identification procedure.

**Method:**

We performed epidural block with the CompuFlo® epidural instrument in 120 healthy women who requested labor epidural analgesia. The epidural needle was considered to have reached the epidural space when an increase in pressure (accompanied by an increase in the pitch of the audible tone) was followed by a sudden and sustained drop in pressure for more than 5 seconds accompanied by a sudden decrease in the pitch of the audible tone, resulting in the formation of a low and stable pressure plateau. We evaluate the sensitivity, specificity, and positive and negative predictive values of the ability of CompuFlo® recordings to correctly identify the true LOR from the false LOR.

**Results:**

The drop in pressure associated with the epidural space identification was significantly greater than that recorded after the false loss of resistance (73% vs 33%) (*P*=0.000001). The sensitivity was 0.83, and the AUC was 0.82.

**Discussion:**

We have confirmed the ability of CompuFlo® to differentiate the false loss of resistance from the true loss of resistance and established its specificity and sensitivity.

**Conclusion:**

An easier identification of dubious losses of resistance during the epidural procedure is essential to reduce the number of epidural attempts and/or needle reinsertions with the potential of a reduced risk of accidental dural puncture especially in difficult cases or when the procedure is performed by trainees.

## 1. Introduction

The loss of resistance technique (LORT) [1] for identifying the epidural space was originally described by Dogliotti in 1933, using saline as a medium and was based on the different densities of tissues encountered as the needle tip passed through the ligamentum flavum into the epidural space. When performing an epidural injection, the following structures are sequentially pierced: the skin, the supraspinous and interspinous ligaments, and then the thick fibrous-elastic ligamentum flavum. According to the original technique, the needle should be inserted until the dorsal aspect of ligamentum flavum: this way the very first loss of resistance is, most likely, the right one.

In clinical practice, however, the epidural needle is most frequently introduced into the lumbar area to a depth of approximately 2-3 cm to avoid accidental epidural space puncture. This way the needle may be located somewhere between the soft tissues, well before the epidural space, and if the LORT with syringe procedure is initiated at this stage, it may give rise to a false loss of resistance.

Most frequently, the false loss of resistance may occur superficial to the epidural space if the needle deviates from the midline and enters the paravertebral muscles. In addition, degeneration of the interspinous ligament with resultant cavity formation has been reported, and needle entry into such a cavity probably accounts for most of the instances of false positive loss of resistance. In that case, the needle has been introduced precisely in the midline [2].

The occurrence of false losses of resistance may be one of the reasons because accurate placement of an epidural needle is one of the more difficult skills that can be mastered by anesthesiologists [3], needing approximately 60–90 placements before reaching an adequate basic skill [4].

Recently, the CompuFlo® instrument (Milestone Scientific Inc. Livingston, NJ, USA) has been introduced to measure the pressure of human tissues in real time at the orifice of a needle [5, 6]. This system is unique; in that, pressure is a feedback loop and a controller to the system, thus regulating the electromechanical motor which controls the flow rate and the fluid dispensed by the system. An audible and visual graphic of exit pressure is provided to focus on the procedure. This instrument has been investigated and validated for epidural use [6, 7].

**Figure 1:**
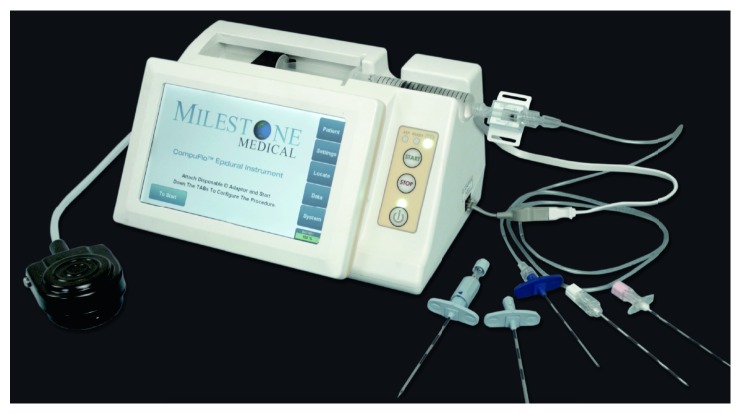
The CompuFlo® epidural instrument.

CompuFlo® has also been reported to be able to differentiate the false loss of resistance due to the location of the epidural needle within the epidural region tissues and the true loss of resistance due to the penetration of the needle into the epidural space [6, 7] (Figure 1), but the sensitivity of this feature is unknown.

For this reason, we undertake this study to investigate the sensitivity and specificity of the ability of CompuFlo® to differentiate the false loss of resistance from the true loss of resistance encountered during the epidural space identification procedure with the LORT (the primary aim of the study).

## 2. Methods

The study received formal approval of the institutional ethics committee. The patient agreed to the referral, and written informed consent was obtained from all participants. We enrolled 120 healthy women with an ASA physical status of I or II between the ages of 20 and 40 years over 38 weeks' singleton gestation, who requested labor epidural analgesia at Casa Sollievo della Sofferenza Hospital (San Giovanni Rotondo) or at Città di Roma Hospital (Rome).

Epidural block was performed in the lateral position using a 16 G Tuohy needle at the L3-L4 or L4-L5 interspace. All the blocks were performed by a senior expert anesthesiologist, and all the CompuFlo® settings and measurements were noted by an independent investigator.

After skin local anesthesia and subcutaneous insertion (2-3 cm) of the Tuohy needle, the device was attached, via 122 cm arterial pressure tubing, to the Tuohy needle in order to register the delta of pressure encountered by the needle during its advancement. The 0 point was made, and the CompuFlo® device was set to deliver normal saline at a rate of 0.05 mL/s with a maximum pressure limited to 120 mmHg. During advancement of the Tuohy needle, pressures were displayed and recorded continuously and the instrument produced an audible tone whose pitch was in accordance with the height of the pressure. According to previous studies [6, 7], a true LOR, indicating the epidural needle has reached the epidural space, was defined as the following pattern: an increase in pressure (accompanied by an increase in the pitch of the audible tone) followed by a sudden and sustained drop in pressure for more than 5 seconds (typically greater than 50% of the maximum pressure) accompanied by a sudden decrease in the pitch of the audible tone, resulting in the formation of a “low and stable pressure plateau.” A false LOR was defined as an increase in pressure followed by a drop in pressure (typically less than 50% of the maximum pressure) that is either not sustained or inconclusive of representing a “low and stable pressure plateau.” If the pressure rapidly increased after a drop in pressure, this was identified as a false loss of resistance, and the operator was elected to continue to advance the needle.

After the entry into the epidural space, the infusion pump was stopped and an epidural catheter was inserted for the intended use.

After the epidural catheter placement, all patients received our routine epidural loading dose (20 mL of levobupivacaine 0.0625% plus sufentanil 10 *µ*g) followed by a programmed intermittent epidural bolus (PIEB) as described elsewhere [8].

The efficacy of epidural block was evaluated by a 100 mm Visual Analogue Pain Scale (0 = no pain; 100 = worst pain ever) assessed at the apex of a painful contraction. Analgesia was considered successful if the patients reported a VAPS equal or less than 10 twenty minutes after administration of the epidural loading dose. Routine follow-up for postanesthetic complications was performed. A successful epidural analgesia rate was recorded.

### 2.1. Statistical Analysis

All the data were recorded and downloaded, and the differences in the pressure drop were compared using the Student's *t*-test.

All the patients who had a plateau pressure remaining below 40 mmHg for at least 5 seconds had a perfect analgesia, and therefore, we assumed this pattern as the reference pattern (true loss of resistance) to evaluate the sensitivity, specificity, and positive and negative predictive values of the ability of CompuFlo® recordings to correctly identify the true LOR from the false LOR.

To evaluate the accuracy of the test, we measured the area under the curve of the receiver operating characteristic (AUC, ROC curve).

The power analysis required a sample size of 115 observations to set 80% test power and 95% significance level.

## 3. Results

All the 120 parturients completed the study, and their data were analyzed. In all cases, epidural block was performed successfully and no complications were noted. Mean (SD) age was 28.3 (6.1) years, with a mean (SD) BMI of 25.7 (1.2) and mean (SD) 39.4 (0.9) weeks of gestation; 74 (61%) patients were nulliparas and 46 (38%) were multiparas.

**Figure 2:**
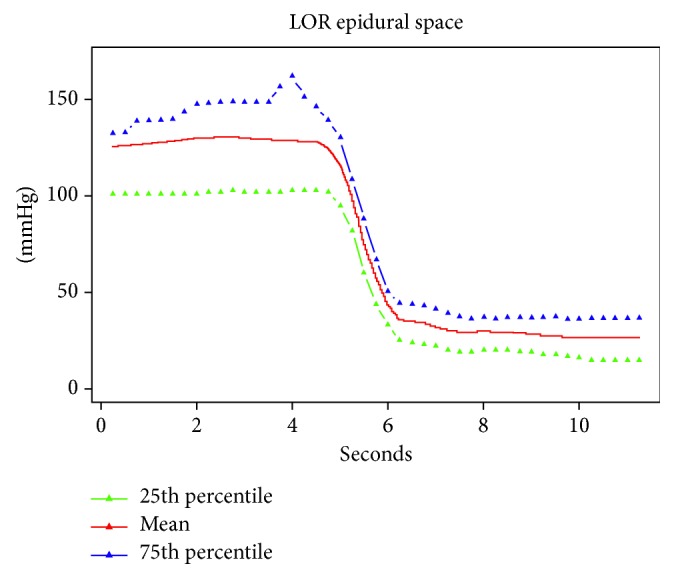
Mean curve (25th and 95th percentile) of the pressure registered by CompuFlo® when the epidural needle was deemed to be in the ligamenta flava and thereafter in the epidural space.

**Figure 3:**
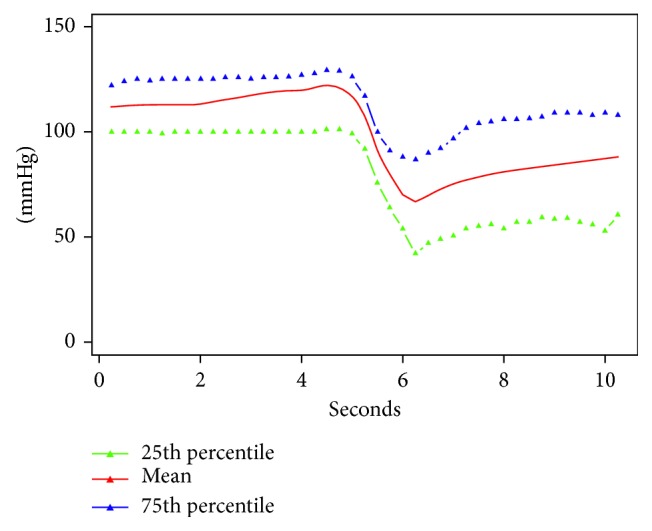
Mean curve (25th and 95th percentile) of the pressure registered by CompuFlo® in the case of false loss of resistance.

**Figure 4:**
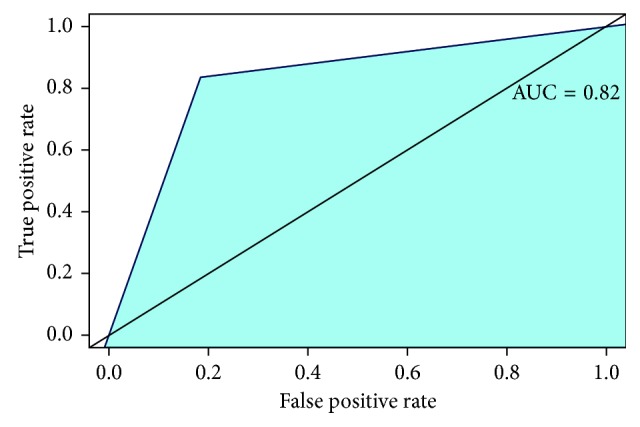
The ROC curve.

In Figure 2 are reported the mean curve (25th and 95th percentile) of the pressure registered by CompuFlo® when the epidural needle was deemed to be in the ligamenta flava and thereafter in the epidural space. In Figure 3 are reported the same curves registered in the case of false loss of resistance. The drop in pressure associated with the epidural space identification (true loss of resistance) was significantly greater than that recorded after the false loss of resistance (73% vs 33%) (*P*=0.000001). In Table 1 are reported sensitivity, specificity, positive predictive value (PPV), negative predictive value (NVP), and the area under the curve (AUC) values.  Figure 4 shows the AUC.

## 4. Discussion

One of the reasons for inadequate or failed epidural block can be due to misidentification of the epidural space with the subsequent malposition of the epidural catheter. A false loss of resistance, in which the tip of the epidural needle lies within subcutaneous, intraligamentous, or paravertebral tissues, is the likely cause of misidentification of the epidural space. This false loss of resistance may occur more likely when the LOR with syringe is attempted before the epidural needle has reached the dorsal aspect of the ligamentum flavum. In addition, if the needle enters the intraspinous ligament at an oblique angle, the needle tip will exit the ligament into the soft tissue on the opposite side with a LOR feel.

The analgesic failure can be usually detected in less than 30 minutes after the initial placement because the patient is still in pain with no evidence of sensory block. However, this late recognition imposes a new block that, in turn, involves a “de novo” procedure that can potentially have newer difficulties and complications. There is no literature investigating this specific aspect of the epidural failure; however, some authors have advocated 17% of failure rates due to the false positive loss of resistance [9].

In a chronic pain management study, an equivalent success rate of 100%, demonstrated by the correct spread of dye during fluoroscopy, was observed in a comparison between the standard LOR epidural technique and CompuFlo® [10]. Other studies in obstetric setting have confirmed the complete analgesic success after the use of CompuFlo® to detect the epidural space [6, 7]. In these latter studies was also hypothesized that this instrument was very useful in helping the anesthesiologist to correctly differentiate the false loss of resistance from the true loss of resistance encountered during the epidural needle advancement during the epidural procedure with the LORT.

In this study, we have confirmed the ability of CompuFlo® and, in addition, we have calculated and established its specificity and sensitivity.

## 5. Conclusions

An important issue linked to an easier identification of dubious losses of resistance during the epidural procedure is that it might lead to a minor number of epidural attempts and/or needle reinsertions with a reduced risk of accidental dural puncture, especially in difficult cases or when the procedure is performed by trainees.

We believe our result may encourage greater use of this device and a more extensive routine evaluation in order to determine its contribution to a simpler and reliable identification of the epidural space not only in terms of efficacy of anesthesia or analgesia but also in reduction of complications.

## Figures and Tables

**Table 1:** Sensitivity, specificity, positive predictive value (PPV), negative predictive value (NPV), and area under the curve (AUC) concerning the ability of CompuFlo® instrument pressure patterns to differentiate the true loss of resistance from the false loss of resistance.

Sensitivity	0.83
Specificity	0.81
PPV	0.72
NPV	0.89
AUC	0.82

## Data Availability

The data used to support the findings of this study are available from the corresponding author upon request.
